# Supplementary material for: Remote heart rate monitoring - Assessment of the Facereader rPPg by Noldus
Source: PLoS One. 2019 Nov 22;14(11):e0225592. doi: 10.1371/journal.pone.0225592 (PMC6874325; doi:10.1371/journal.pone.0225592)
Supplement: Attachment — Submitted filename: Response to Reviewer_16489.docx [file pone.0225592.s002.docx]

Treviso (Italy), June 10^th^, 2019

**Rebuttal letter**

PONE-D-18-36091
Remote heart rate monitoring - Assessment of the Facereader^TM^ rPPg by Noldus
 **Reviewer #1**

**R#1.** *The introduction provides an in-depth summary of rPPG but does not explicitly identify problem/s or research question/s that the study was designed to address. The introductory content should establish a clear knowledge gap/rationale that underpins specific research questions. Given this lack of clear rationale, the introduction appears unnecessarily lengthy.*

**Authors.** We agree with the reviewer and we have modified the introduction accordingly. We had better state problems or research questions the study was designed to address. In this sense we have add that our validation, in case of significant results, could assume a strong relevance regarding the potential but realistic application of this technology in workplace environments, where, for example, it is possible to find conditions (lighting conditions, position and movements of the person, distance from the screen and so on) very similar to those in which took place the entire experimental procedure.

**R#1.** *Can the authors descriptively summarise the HR data across rest and stress phases of the experimental protocol? A tabular format may be most appropriate. E.g. mean/SD/range heart rates together with mean biases/CI/LoA for each for each condition.*

**Authors**. These kinds of parameters were not part of the research objectives or the researchers interests. The objective of the study was to assess the accuracy of a consumer rPPG system with respect to HR monitoring and compare its performance to the gold standard ECG collecting a large amount of data, spanning the widest possible range of HR frequencies and we have not catalogued HR data across the stress/rest phases. We feel that the Bland –Altman plots (figures 3 and a) show clearly the mean differences and LoA across the HR data.

**R#1.** *Can the authors add different shading to figures 3 and 4 to show rest and stress measures? This simple change would enable more in-depth interpretation of biases.*

**Authors.** As before, we have not catalogued HR data across the stress/rest phases and as we stated in the conclusions “Although the Facereader^TM^ rPPG revealed a mean bias of just 9.8 bpm compared to an ECG criterion measure, our results show the agreement between the Facereader rPPG and the ECG is poor. However, the mean bias is highly influenced by the fact that the Facereader tends to over-estimate lower heart rates, and under-estimates higher heart rates compared to the ECG and the error ranges from almost -30 up to +50 bpm.”

**R#1.** *Have the authors considered other parameters to elucidate measurement error such as the coefficient of variation or standard error of measurement (NB: not standard error of the mean)?*

**Authors.** The coefficient of variation is more appropriate for data that follow a lognormal distribution, such as biomarker concentrations in the blood. The %CV presents the variation in the measurement as a percentage of the mean. This is only appropriate where variation increases in proportion to the measurement, which you only get with lognormal data. Our presentation is equivalent, in that we show the variation in the measure as a difference from the mean.

The standard error of the measurement is the standard deviation of the observed heart rate for a given true score. That is essentially what the Bland-Altman plot presents, but the plot is better in presenting more interpretable limits of agreement (+-2 SDs) and by using Oldham’s method for plotting difference vs average, is unlikely to mislead by suggesting an association where there is none. It has previously been shown that Bland-Altman’s approach is just as appropriate where one method is a gold standard as when both are measured with error.

We do also present the intraclass correlation coefficient (ICC) as an alternative unitless parameter to describe the extent of the measurement error.

**R#1.** *L168/69: please describe positions of the electrodes.*

**Authors**. Although the position of the electrodes is briefly described in lines 168-169 and could be deduced from the figure on the experimental set-up, we have added that the electrodes were applied in the second standard derivation according to the Einthoven triangle and in a proximal position (Line 182-184 of the reviewed document).

**R#1.** *L195: The introduction mentioned advances in data processing to handle varying skin tones yet the authors recruited an all-Caucasian sample. Can the authors explain why they chose not to include more heterogeneous ethnicities in their sample? Is this noted in the study limitations?*

**Authors**. Including this variable (skin tone) was not among the objectives of the study and would have required different control methods and analysis. We have added this point as a possible limitation of the study.

**R#1.** *Table 1: inconsistent use of decimal places. E.g. some means have 1 dp but SDs are integers and vice versa. Some ranges have 1 dp at one end but not at the other.*

**Authors.** We agree with the reviewer and we have edited the manuscript for consistency.

We had standardized the table, which shows averages and d.s. of the characteristics of the sample, approximating all the values to whole units.

**R#1.** *L271-73: this statement could be used as part of a more complete rationale in the introductory section.*

**Authors.** We thank the reviewer for pointing this out and we have modified the manuscript accordingly.

**Reviewer #2**

**R#2.** *The authors did not provide information in the manuscript on: About features of the illumination pulsation. In the technical manual of the Extech 403125 digital light meter (www.extech.com) these features were not mentioned (pulsations of the Computer's Monitor illumination and its influence on the illumination of the study object (face of the individual) cannot be excluded. In manuscript there is no mention of proof of influence (or lack of it) on total illumination of the object).*

**Authors**. Both the ambient (illumination) and screen (luminance) were controlled and kept constant during the entire experiment. These parameters were assessed by a digital light meter pointed towards the screen.

The variations in the illumination of the screen due to the alternation of the stimuli of the tasks are not impacting for the purpose of detecting the signal from the face of the participants, both for the distance between the participant's face and the screen (about 60 cm) and for the size of the stimuli with respect to the presentation screen.

**R#2.** *About individual movements of study object. Providing continuous spatial monitoring of objects for 20 minutes is impossible, therefore, it is necessary to control the movement of objects using hardware or software means.*

**Authors**. We agree with the reviewer that movements from the participant may causes artefacts in the HR monitoring and we have carefully removed any motion-induced artefacts from the data analysis.

**R#2.** *On the procedure for the formation of instantaneous heart rate values to support the procedure for downgrading data to a frequency of 8 Hz. This is desirable to do, since the cardio signal is a temporary discrete series, and the average value of the pulse is about the order of 1 Hz.*

**Authors**. We agree with the reviewer and we have modified the manuscript accordingly specifying that the data downgrading was carried out using the BioGraph software which, among its features, allows reliably performing such data processing.

**Reviewer #3**

**R#3.** *Despite the authors say that no previous study was published on the topic, except the one of the owners of the FaceReader device, I see there is a recent study that appeared on MDPI Sensors (https://www.mdpi.com/1424-8220/18/6/1802/htm).*

**Authors**. We thank the reviewer for pointing us to this study. We apologize for not referencing this work and we have included a citation in the revised manuscript.

We apologise for missing this recent paper published after we started drafting our own manuscript, but before we completed submission. We are happy to update our introduction to refer to it. However, the study differs from our own in the following ways:

- the research objectives and study assumptions are different;
- the experimental procedure, the methodology and data analysis are different;
- the focus of the other study was not the validation of the FaceReader as a commercially available tool capable of remotely measuring HR, but comparing different solutions, methodology (including for example ML-machine learning), and heart rate monitoring tools.

However, whilst we do not feel the prior publication detracts from the novelty of our own work, we note PloS ONE’s editorial policy of evaluating submitted manuscripts on the basis of methodological rigor, regardless of perceived novelty. We argue that our methods are of a high standard, and in many regards better than this previously published article, which may to some extent explain the different conclusions.

**R#3.** *The authors provide a comparison between two commercial solutions. However, nothing is said about the algorithms behind these tools. For instance, in the ProComp Infiniti System used for the ECG, it is said that the internal routines were used for HR extraction and then that "Since the Facereader rPPG allows to process data at 8 Hz, the ECG criterion measure data were downgraded to 8 Hz" but no detail is provided on the original sampling rate (of both ECG and HR signal), on the characteristics of the ProComp HR estimation algorithm (instantaneous, averaged over time) etc.*

**Authors**. Procomp Infiniti is a professional tool used in the clinical and experimental field (for example in biofeedback training and therapy) and constitutes a gold-standard for the measurement of physiological signals. The Procomp has been employed in more than 300 (published) experimental studies, including our previous study on the assessment of the Fitbit Charge 2 for monitoring heart rate, published in PlosOne one year ago. (Benedetto, S., Caldato, C., Bazzan, E., Greenwood, D. C., Pensabene, V., & Actis, P. (2018). Assessment of the Fitbit Charge 2 for monitoring heart rate. PloS one, 13(2), e0192691).

**R#3.** *Finally, the statistical analysis appears incomplete to me, since no R square data were reported and, in the presence of the trend in the Bland-Altman plots, there is no data transformation to correct the trend.*

**Authors.** The R-Squared statistics presents the percentage of variation in an outcome explained by the predictors in a regression model. Regression is not an appropriate method for assessing how well a measurement tool agrees with a gold standard because it takes no account of whether the predicted HR are close to the true HR. If all values were under-estimated by 50bpm, the R-squared would still be 100%, inappropriately suggesting agreement where none existed. It is the same argument for why correlation is inappropriate. The presence of the trend in teh Bland-Altman plots is not something to be transformed away, but a feature of the disagreement to be estimated. The trend indicates that low HR are over-estimated and high HR are under-estimated, as is also clear from figure 2. Whatever method is used, it should be very clear that the Facereader results have little resemblance to the actual HR in our study and are little better than random noise.
